# Feasibility of a prison-based test-and-treat model for enhancing hepatitis C care in Kedah, Malaysia

**DOI:** 10.1186/s12889-025-22296-0

**Published:** 2025-03-26

**Authors:** Mohd Azri Mohd Suan, Ahmad Muaz Zulkifli, Noor Hidayati Ani, Azlina Azlan, Mohamad Faiz Mustafa, Siti Maisarah Md Ali, Siti Aishah Aris, Nur Aisyah Sakinah Muhamad Nizar, Fatihah Fahami Mohd Najib Fahami, Amizah Othman, Huan-Keat Chan, Muhammad Radzi Abu Hassan

**Affiliations:** 1 Clinical Research Center, Institute for Clinical Research, Hospital Sultanah Bahiyah, Ministry of Health Malaysia, Alor Setar, Kedah Malaysia; 2 Health Clinic, Pokok Sena Prison, Pokok Sena, Kedah Malaysia; 3 Health Clinic, Alor Setar Prison, Alor Setar, Kedah Malaysia; 4 Public Health Division, Kedah State Health Department, Ministry of Health Malaysia, Alor Setar, Kedah Malaysia; 5 Microbiology Unit, Pathology Department, Hospital Sultanah Bahiyah, Ministry of Health Malaysia, Alor Setar, Kedah Malaysia; 6 Office of Director General, Ministry of Health Malaysia, Putrajaya, Malaysia

**Keywords:** Antiviral agents, Health services accessibility, Hepatitis C, Malaysia, Prisons

## Abstract

**Background:**

Hepatitis C virus (HCV) infection remains a public health concern, significantly affecting vulnerable populations including people who use drugs and in prisons. This study assessed the feasibility of a new prison-based test-and-treat model for HCV in two prisons in Kedah, Malaysia.

**Methods:**

The model was tested on participants newly admitted between June 2022 and December 2023 to one of the two selected prisons, one for pretrial detention and the other for serving sentences. It features a streamlined test-and-treat procedure within prisons, encompassing anti-HCV (exposure) testing, HCV ribonucleic acid (RNA) (current infection) testing, pretreatment assessments, and treatment initiation. The outcome assessment focused on (I) screening coverage, (II) implementation success across the HCV care cascade, ranging from anti-HCV detection, HCV RNA confirmation, treatment initiation, and completion to a sustained virological response rate and (III) practicality within resource-limited prison environments.

**Results:**

All 18,811 (100%) newly admitted participants were screened for HCV during the study period, with 4,054 (21.6%) of them testing positive for anti-HCV (HCV exposure). A total of 793 (19.6%) participants underwent HCV RNA testing, with 655 (82.6%) confirmed to have HCV infection. Those with HCV RNA test results were mainly male (98.1%), with nearly half (48.2%) aged 40–49 years. A vast majority (95.7%) reported at least one HCV risk factor, primarily injection drug use (95.2%). Of the 655 participants with a positive HCV RNA test, 648 (98.9%) completed pretreatment assessments. Antiviral treatment was initiated in 319 (49.2%) of them, with 165 (51.7%) completing the full course. Among those who completed treatment, 80 (48.5%) underwent HCV RNA testing 12 weeks after treatment, with 77 (96.3%) achieving a sustained virologic response.

**Conclusion:**

This new prison-based test-and-treat model for HCV infection demonstrates promising feasibility, as indicated by high screening coverage and successful implementation across the HCV care cascade using existing resources. These findings suggest the potential for broader adoption of this model in correctional facilities. Further research is needed to improve treatment completion and address factors contributing to dropout.

## Background

Hepatitis C virus (HCV) infection remains a significant global public health concern, with an estimated 50 million people living with chronic infection worldwide [1]. This places a substantial burden on healthcare systems [2]. Chronic HCV infection can lead to serious liver complications, such as cirrhosis and hepatocellular carcinoma (HCC) [3], ultimately contributing to significant morbidity and mortality [4, 5].

Recognising the urgency of addressing this global challenge, the World Health Organization (WHO) launched the Global Health Sector Strategy for Hepatitis in 2016 [6]. This ambitious strategy aims to eliminate viral hepatitis, as a public health threat by 2030. Recent advancements in treatment and diagnostics, coupled with significant cost reductions in both affordable treatment and diagnostic tests, have transformed the global response to HCV elimination. These advancements include highly effective direct-acting antiviral (DAA) therapy [7, 8], point-of-care serological and nucleic acid testing for HCV [9, 10], and evidence-based guidelines for HCV testing and treatment [11]. These developments have paved the way for large-scale expansion of HCV test-and-treat programs worldwide.

Building on this global momentum, Malaysia has firmly committed to achieving the WHO goal of eliminating viral hepatitis. In working towards achieving this commitment, the government has launched the 5-year National Strategic Plan for Hepatitis B and C, outlining strategies to enhance accessibility of HCV care for vulnerable populations facing barriers to conventional hospital-based care [12], which includes any diagnostic activities or treatment care related to hepatitis C provided at public hospitals. A key strategy in this national agenda involves a microelimination approach through systematic screening and treating HCV-infected vulnerable groups, such as those who are in prisons and drug rehabilitation centers [12].

Prison settings present a unique challenge. People who are in prisons have a higher risk of contracting HCV compared to the general population, mainly due to primary risk factors such as injection drug use (IDU) and unsafe tattooing or equipment sharing [13]. This elevated risk translates into a high HCV burden within prisons, potentially undermining the health and economic benefits achieved through the expansion of DAA therapy in the community. Therefore, it is important to explore innovative approaches to enhance access to HCV care within correctional settings. While the effectiveness of HCV elimination programs in the general population is well established [14–17], there are limited data on their feasibility for vulnerable populations, particularly those who are in prisons. Furthermore, successful microelimination programs have been implemented in prison settings in various countries, demonstrating the feasibility of achieving high treatment rates and reducing HCV transmission [18, 19].

To address this knowledge gap and explore the potential of HCV elimination initiatives in prison settings, an inter-ministerial collaboration has been established between the Ministry of Health and the Ministry of Home Affairs. A new test-and-treat model was tested on people who are in one of the two selected prisons in Kedah state, with support of a nearby hospital. This study aimed to evaluate the feasibility of this model in improving HCV care within prison settings.

## Methods

### Study design

This study employed a retrospective observational cohort design. Data were retrospectively retrieved and analysed from records collected during the implementation of a prison-based test-and-treat model for Hepatitis C Virus (HCV) in two prisons in Kedah, Malaysia, between June 2022 and December 2023. At the time of data collection, the information was gathered as part of routine health care service delivery.

### Study setting

A study team from the Hospital Sultanah Bahiyah, led by a physician, implemented a new test-and-treat model in the two selected prisons located in Kedah state. For this study, of the three prisons located in Kedah state, the juvenile prison was excluded, and the two adult prisons were chosen to implement the HCV test-and-treat model, as the model focused on eliminating HCV in adult participants. The Pokok Sena Prison primarily housed sentenced individuals, both male and female prisoners, with a capacity of 2,300 adults. Female prisoners constitute less than 20% of the total prison population in Pokok Sena Prison. Alor Setar Prison is a male-only prison, primarily for pretrial detention, with a capacity of 1,750 adults. Harm reduction services, such as opioid agonist therapy (OAT) and needle and syringe programs (NSP), are not currently available in both prisons. Although HCV screening and treatment services (HCV antibody testing, RNA testing, and direct-acting antiviral (DAA) treatment) are available outside the prisons at government primary health clinics and hospitals, neither prison had the capacity to provide similar HCV care within the prisons. This is due to the prison’s high occupancy rate and the small resident medical team of five members each in both prisons, as well as ongoing resource constraints and logistical challenges, which existed before and throughout the study period.

### Implementation of the prison-based test-and-treat model

**Fig. 1:**
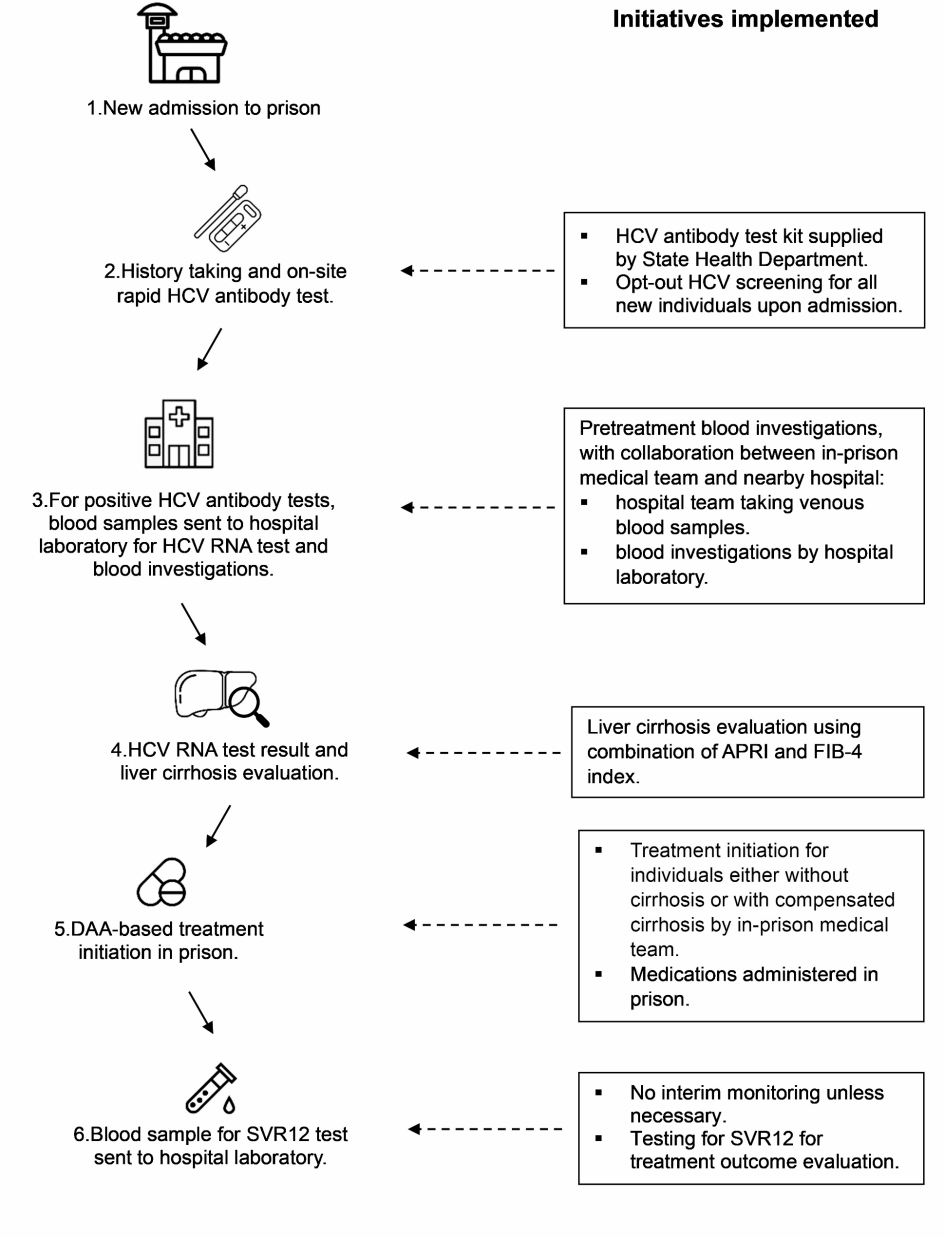
New prison-based test-and-treat model for prisons in Kedah state

The prison-based test-and-treat model was adapted from the Clinical Practice Guideline for Management of Chronic Hepatitis C in Malaysia [20]. This model, developed collaboratively by the Ministry of Health and the Ministry of Home Affairs, streamlined the procedure of identifying and treating HCV infection among people who are in prisons (Fig. 1). Before its implementation, hepatitis C screening was not routinely conducted in prisons. While HIV and tuberculosis screening are standard procedures, HCV testing was previously limited to individuals who showed symptoms of liver disease, disclosed recent risk factors to prison health officers, or had coinfections such as HIV.

After implementing this model, all newly admitted participants to prisons underwent a thorough history taking and screening process conducted by the in-prison medical team, including an on-site rapid HCV antibody test as an additional procedure. The HCV antibody test kits were supplied by the State Health Department to prisons. The model utilised an opt-out approach, where all newly entered prisoners were offered the screening test, which was performed unless explicitly declined. In cases where a positive HCV antibody test yielded a positive result, a confirmatory HCV RNA test was performed, along with comprehensive blood tests to assess treatment eligibility (pretreatment assessment) including a platelet count, aspartate aminotransferase (AST) and alanine transaminase (ALT) levels, during the same prison clinic visit.

The study team collaborated with the in-prison medical team, visiting the prison clinic on a scheduled plan, approximately 4–5 times per month, and assisted with blood sample collection, sample labelling and management, and provided brief health education to participants. Subsequently, the study team helped transport the samples to the hospital pathology lab and actively tracked lab results. Upon availability, all blood test results were promptly entered and shared with the prison team via a password-protected central database, facilitating a smoother transition into treatment compared to the previous standard of care. Prior to this model, the in-prison medical team, with limited workforce, faced challenges in tracing lab results promptly, leading to potential delays in treatment initiation. Following HCV RNA test, participants with complete pretreatment assessment will have their liver fibrosis assessment using a sequential combination of AST-to-Platelet Ratio Index (APRI) and the Fibrosis-4 (FIB-4) index to determine the presence and severity of cirrhosis [21]. Participants with an APRI score of 1.5 or greater were classified as having a high probability of cirrhosis. For participants with an APRI score between 1.0 and 1.5, a FIB-4 score was subsequently calculated. Those with a FIB-4 score of 3.25 or greater were also classified as having a high probability of cirrhosis. Participants with an APRI score less than 1.0 were classified as no cirrhosis [21]. This sequential approach, utilising APRI as a primary screening tool followed by FIB-4 for intermediate cases, is a pragmatic strategy to facilitate DAA treatment in a resource-limited setting.

In addition to the APRI and FIB-4 assessment, the in-prison medical officer performed a clinical assessment to check for any signs of liver decompensation before initiating DAA treatment. Participants with a high probability of cirrhosis based on the sequential approach, and have suspicious signs of liver decompensation, were referred to a gastroenterologist at the hospital for further assessment. Likewise, those with coinfections such as HIV and HBV were referred to hospital for specialised gastroenterology care. Participants with no cirrhosis or compensated cirrhosis received treatment with a DAA-based regimen, initiated by in-prison medical officers. The treatment duration varied based on cirrhosis status, with 12 weeks for participants without cirrhosis and 24 weeks for participants with compensated cirrhosis. DAAs were supplied by the hospital pharmacy to prisons and dispensed in full to all eligible participants at the start of treatment. This ensured that all participants had access to the complete treatment regimen, regardless of their release or transfer status. Although routine monitoring, which includes laboratory investigations and toxicity monitoring (liver function test, creatinine) at 4 and 12 weeks following DAA treatment initiation as required by the National Clinical Practice Guideline [20], is not routinely needed, this model aims for SVR testing 12 weeks after treatment completion (SVR12). Blood samples were sent to the hospital laboratory for SVR12 testing. Those who did not achieve an SVR were referred for specialised gastroenterology care in hospital.

### Outcome and feasibility assessment

Similar to the methods used by Markby et al. [14] and Hassan et al. [22], this study evaluated the feasibility of this new test-and-treat model across three key areas; (I) demand, by assessing the screening coverage, (II) implementation success rate, by evaluating the achievement in the HCV care cascade, ranging from anti-HCV detection, HCV RNA test, treatment initiation, completion to SVR12, and (III) practicality, by assessing the ability to confirm HCV diagnosis and initiate treatment timely despite resource constraints within prisons.

Specific outcomes were measured as proportions of participants who; (I) underwent HCV antibody testing upon admission, (II) underwent HCV RNA testing, (III) received a valid HCV RNA test result, (IV) tested positive for HCV RNA, (V) received DAA-based treatment within the prison, (VI) completed treatment, (VII) underwent SVR12 testing, and (VIII) achieved SVR12. Additionally, this study measured the time interval between HCV RNA testing and treatment initiation.

### Data collection and analysis

Only limited information was gathered for newly admitted prison population who underwent HCV screening test between June 2022 and December 2023 due to lack of standardized data collection procedures and the data was not collected with the intention of research at the point of initial collection. During model implementation, data collection was primarily focused on participants with positive screening test and had confirmatory HCV RNA testing. Data were retrospectively retrieved by the same team that implemented the care model, from records collected at the prison facilities during the implementation of the HCV test-and-treat model. These records consolidated into a central database and included demographics, history of exposure to risk factors for HCV infection, blood investigation results including liver function tests, date of HCV screening, HCV ribonucleic acid (RNA) results, date of treatment initiation and completion, and test results for sustained virologic response 12 weeks after treatment (SVR12). Participants with prior experience with DAA treatment were excluded from the data analysis. These participants were referred to a gastroenterologist at the hospital for second-line DAA treatment, which was outside the scope of this prison-based model. Therefore, their data were not collected.

All data were deidentified before analysis to ensure participant confidentiality. The statistical analysis was performed using the R statistical software version 4.3.2 [23]. Descriptive statistics were used to summarise participant characteristics and outcomes of test-and-treat model implementation. Categorical variables, including demographics and risk factors for HCV, were reported as frequencies and percentages. Numerical variables, including age and blood investigation results, were reported as means and standard deviations (SDs) for normally distributed data, and as medians and interquartile ranges (IQRs) for skewed data. The durations between steps in the care cascade was reported as medians and IQRs. A comparison of the duration of cascade steps between two prisons was conducted using the Mann-Whitney test.

The proportions of participants who underwent onsite HCV antibody and HCV RNA testing were determined. Analysis of HCV RNA status was conducted only among participants who underwent HCV RNA testing. Treatment initiation was analysed in those who tested positive for HCV RNA, while treatment completion was assessed in those who received treatment. SVR assessment was determined for those who completed treatment and underwent testing.

## Results

### Study population characteristics

During the study period, all 18,811 (100%) participants newly admitted to prisons were screened. None of the newly entered prisoners declined the testing. Of this, 4,054 (21.6%) tested positive for HCV antibodies, and 793 (19.6%) participants underwent confirmatory testing using HCV RNA. The observed dropouts between anti-HCV positive and HCV RNA testing were due to limited workforce capacity within prison setting and participant unavailability. Among 793 participants who were anti-HCV positive and underwent confirmatory testing, their mean age was 42.4 years (SD = 7.67), with nearly half (48.2%) aged between 40 and 49 years. The majority were male (98.1%) and reported at least two risk factors for HCV (95.7%), most commonly injection drug use (95.2%) and prior incarceration (77.3%). Liver function tests yielded an average AST level of 40.9 U/L (SD = 24.49) (normal range: 5–34 U/L) and an ALT level of 55.1 U/L (SD = 51.47) (normal range: 0–55 U/L). The average platelet count was 273.0 × 10^3/uL (SD = 83.77) (normal range: 176–407 × 10^3/uL). Only one participant had hepatitis B coinfection, and none had HIV coinfection. Characteristics of participants with anti-HCV positive and underwent confirmatory tests are summarised in Table 1.

**Table 1:** Characteristics of participants with anti-HCV positive and underwent confirmatory testing

Characteristics		Total	(% total)	PAS (% participants from PAS)		PPS (% participants from PPS)	
**Overall**		793	(100)	412	(100.0)	381	(100.0)
**Age (mean**,** SD)**		42.4	(7.67)	42.6	(7.43)	42.1	(7.92)
**Age group**							
	< 30	44	(5.5)	16	(3.9)	28	(7.3)
	30–39	232	(29.3)	119	(28.9)	113	(29.7)
	40–49	382	(48.2)	202	(49.0)	180	(47.2)
	50–59	125	(15.8)	72	(17.5)	53	(13.9)
	≥ 60	10	(1.3)	3	(0.7)	7	(1.8)
**Gender**							
	Male	778	(98.1)	412	(100.0)	366	(96.1)
	Female	15	(1.9)	0		15	(3.9)
**Risk factor** ^**±**^							
	People who inject drugs ^§^	755	(95.2)	395	(95.9)	360	(94.5)
	History of invasive medical procedures	53	(6.7)	0		53	(13.9)
	Recipient of blood/blood products before 1994	1	(0.1)	0		1	(0.3)
	Tattooing	183	(23.1)	16	(3.9)	163	(42.8)
	Body piercing	53	(6.7)	3	(0.7)	50	(13.1)
	Partner who is HCV- infected	1	(0.1)	0		1	(0.3)
	Men who have sex with men	21	(2.7)	0		21	(5.5)
	Previously in jail/prison	613	(77.3)	318	(77.2)	295	(77.4)
	Sex worker	37	(4.7)	2	(0.5)	35	(9.2)
**Number of risk factors**							
	1	18	(2.3)	13	(3.2)	5	(1.3)
	2–4	759	(95.7)	398	(96.6)	361	(94.8)
	> 4	16	(2.0)	1	(0.2)	15	(3.9)
**Blood results (mean**,** SD)**							
	AST (U/L)	40.9	(24.49)	37.2	(22.69)	45.0	(25.75)
	ALT (U/L)	55.1	(51.47)	54.4	(51.47)	55.9	(51.53)
	Platelet (x 10^3/uL)	273.0	(83.77)	276.1	(89.92)	269.6	(76.45)

^±^ Participant can have more than one risk factor. Percentages are calculated using the total number of participants in each prison as the denominator.

^§^ People who inject drugs: Participants with any history of injection drug use (past or present)

Abbreviations: SD: standard deviation; PAS: Alor Setar Prison; PPS: Pokok Sena Prison; AST: aspartate aminotransferase; ALT: alanine transaminase

### Outcomes of model implementation

**Fig. 2:**
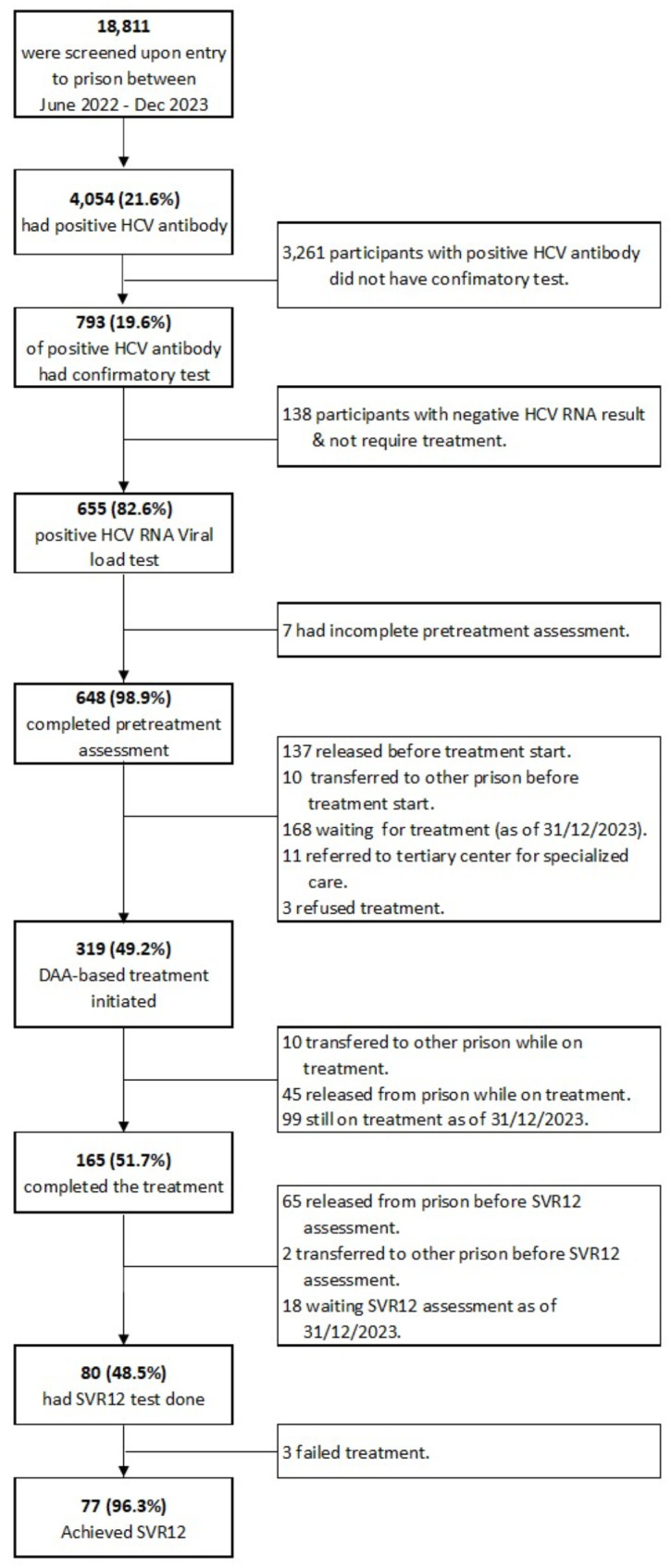
Progression of participants newly admitted to prison through HCV care cascade in a prison-based test-and-treat model, Jun 2022– Dec 2023

Among the 793 participants who were HCV antibody positive and underwent confirmatory testing, 655/793 (82.6%) tested positive for HCV RNA. Among them, 648/655 (98.9%) completed pretreatment assessments which includes platelet count, AST levels, and ALT levels. Subsequently, 319/648 (49.2%) initiated treatment, and 165/319 (51.7%) completed the treatment. Following treatment completion, 80/165 (48.5%) underwent SVR12 assessment, and 77/80 (96.3%) participants achieved SVR12 (Fig. 2). Three participants who did not achieve SVR12 were referred to specialised gastroenterology care in hospital for further evaluation and management.

Of the 648 participants who tested positive for HCV RNA and completed the pretreatment assessments, 625/648 (96.5%) did not have cirrhosis (APRI score ≤ 1.0) and 23/648 (3.5%) had cirrhosis based on the sequential approach (APRI ≥ 1.5 or FIB-4 ≥ 3.25 for those with APRI between > 1.0 and < 1.5). Treatment was initiated in 313/625 (50.1%) non cirrhotic participants. One participant refused treatment, one was referred to the hospital due to hepatitis B coinfection, 132/625 (21.1%) were released from prison before treatment initiation, 10/625 (1.6%) were transferred to another prison, and 169/625 (27.0%) were waiting for treatment as of 31 December 2023. Of the 23 participants with cirrhosis, treatment was initiated for 6/23 (26.1%) participants. Ten (43.5%) were referred to the tertiary hospital for specialised care due to suspected decompensated cirrhosis, 5/23 (21.7%) were released before treatment initiation, and 2/23 (8.7%) refused treatment.

Among those who received treatment, the majority (293/319, 91.8%) received 12-week treatment with sofosbuvir-daclatasvir, while 20/319 (6.3%) received 12-week treatment with sofosbuvir-ravidasvir. Additionally, 6/319 (1.9%) compensated cirrhotic participants received 24-week treatment with sofosbuvir-daclatasvir (Table 2). No serious adverse events were reported among treatment recipients. Participants at Alor Setar Prison were more likely to receive treatment (57.8% vs. 37.9%; *p* = 0.024) compared to those serving sentences at Pokok Sena Prison. No other significant differences in the care cascade between the two prisons.

**Table 2:** Outcomes of participants with anti-HCV positive and underwent HCV confirmatory testing stratified by type of prison

Outcomes	Overall *n* (%)		PAS *n* (%)		PPS *n* (%)		*p*-values for PAS vs. PPS
**HCV antibody positive**	4054		2340		1714		
**Underwent confirmatory test**	793/4054	(19.6)	412/2340	(17.6)	381/1714	(22.2)	
**HCV RNA test result**							0.822^a^
Positive	655/793	(82.6)	342/412	(83.0)	313/381	(82.2)	
Negative	138/793	(17.4)	70/412	(17.0)	68/381	(17.8)	
Invalid	0/793						
**Pretreatment assessment**							0.059^b^
Complete	648/655	(98.9)	341/342	(99.7)	307/313	(98.1)	
Incomplete	7/655	(1.1)	1/342	(0.3)	6/313	(1.9)	
**APRI Score**							0.488^a^
≤1.0	576/648	(88.9)	302/341	(88.6)	274/307	(89.3)	
Between > 1.0 and < 1.5	48/648	(7.4)	28/341	(8.2)	20/307	(6.5)	
≥1.5	24/648	(3.7)	11/341	(3.2)	13/307	(4.2)	
**FIB-4 Score** ^ǂ^							0.998^b^
<3.25	46/48	(95.8)	27/28	(96.4)	19/20	(95.0)	
≥3.25	2/48	(4.2)	1/28	(3.6)	1/20	(5.0)	
**Cirrhosis status**							0.495^a^
Cirrhotic	26/648	(4.0)	12/341	(3.5)	14/307	(4.6)	
Non-cirrhotic	622/648	(96.0)	329/341	(96.5)	293/307	(95.4)	
**Referred to the tertiary hospital for specialised care**	11/648	(1.5)	11/341	(3.2)	0/307	(0)	
**Received DAA-based treatment**	319/648	(49.2)	197/341	(57.8)	122/307	(39.7)	0.024^a^
**Treatment duration and regimen**							
12-week of sof/dac	293/319	(91.8)	196/197	(99.5)	97/122	(79.5)	
12-week of sof/rav	20/319	(6.3)	1/197	(0.5)	19/122	(15.6)	
24-week of sof/dac	6/319	(1.9)	0/197	(0)	6/122	(4.9)	
**Completed treatment**	165/319	(51.7)	99/197	(50.3)	66/122	(54.1)	0.581^a^
**Had an SVR12 test done**	80/165	(48.5)	36/99	(36.4)	44/66	(66.7)	0.002^a^
**SVR12 test result**							0.585^b^
Achieved SVR12	77/80	(96.3)	34/36	(94.4)	43/44	(97.7)	
Failed treatment	3/80	(3.8)	2/36	(5.6)	1/44	(2.3)	

^a^ Pearson’s Chi-square test, ^b^ Fisher’s exact test.

^ǂ^ FIB-4 scores were calculated for 48 participants with APRI scores between > 1.0 and < 1.5.

Abbreviations: PAS: Alor Setar Prison; PPS: Pokok Sena Prison; HCV: hepatitis C virus; SVR 12: sustained virologic response 12-week post-treatment; DAA: direct-acting antiviral; sof/dac: sofosbuvir/daclatasvir; sof/rav: sofosbuvir/ravidasvir

The median time to collect blood samples for HCV RNA after a positive antibody test was 8 days, consistent across both prisons (Table 3). However, differences emerged in subsequent steps. Participants at Alor Setar Prison received their RNA test results in 11 days, faster than those at Pokok Sena Prison did (15 days, *p* < 0.05). Conversely, participants at Pokok Sena Prison experienced a shorter wait to initiate treatment after a positive RNA test (37 days vs. 42 days; *p* = 0.020). The median time to complete the entire testing and treatment initiation process was 67 days, with no significant difference between the two prisons (*p* = 0.482).

**Table 3:** Time to events for steps in the HCV care cascade stratified by type of prison

Prison	HCV antibody testing - RNA specimen collection			RNA specimen collection - RNA result available			RNA result available - DAA treatment initiation			Total: HCV antibody testing - DAA treatment initiation		
	*n*	Med (days)	IQR	*n*	Med (days)	IQR	*n*	Med (days)	IQR	*n*	Med (days)	IQR
Overall	793	8	7–15	793	14	7–19	319	40	24–58	319	67	50–85
PAS	412	8	6–14	412	11	6–16	198	42	29–53	198	66	56–76
PPS	381	8	8–21	381	15	8–22	121	37	15–63	121	73	44–100
*p*-values for PAS vs. PPS ^a^		< 0.05			< 0.05			0.020			0.482	

^a^ Mann-Whitney test

Abbreviations: PAS: Alor Setar Prison; PPS: Pokok Sena Prison; HCV: hepatitis C virus; DAA: direct-acting antiviral; Med: Median; IQR: inter-quartile range

## Discussion

This study demonstrates a promising approach to identify and treat HCV infection in prison settings. The collaborative effort among public health officials, hospital clinicians, and in-prison medical staff has proven effective in tackling HCV within this vulnerable population. The model adapts a standard test-and-treat approach, demonstrating its feasibility for detecting HCV and initiating antiviral treatment in prisons. More importantly, this approach optimises existing government resources, ensuring financial viability for broader implementation. This contrasts with strategies in prisons in developed countries, which often involve more resource-intensive methods such as point-of-care HCV RNA testing or transient elastography for liver stiffness assessment [24, 25]. By leveraging existing resources, this model has the potential to be adopted in resource-constrained correctional settings, particularly in low- and middle-income countries (LMICs).

This prison-based model differs from traditional targeted screening practices for HCV, which can stigmatise and discriminate against high-risk participants [26]. It integrates HCV screening with other routine entry tests for all participants newly admitted to prisons [27]. This strategy significantly increased testing and detection rates for HCV, as evidenced by the identification of over 4,000 participants who tested positive for HCV antibodies during the study period. Similar approaches have previously been successfully implemented in California and Australia [28, 29]. In Malaysia, HCV testing can be easily bundled with HIV and tuberculosis testing, thereby streamlining the screening process in prisons. Nonetheless, despite many participants testing positive for HCV antibodies, only about 20% underwent subsequent confirmatory testing. This bottleneck was attributable to the limited capacity of medical staff in prisons to conduct confirmatory RNA testing. As described in the study setting, the prisons faced high occupancy rates, and each prison had a small resident medical team of only five members. This resource constraint significantly hampered the ability to provide timely RNA testing to all HCV antibody-positive participants. Furthermore, participant unavailability due to release or transfer to other prisons also contributed to this issue. As demonstrated in Table 3, the median time from HCV antibody testing to RNA specimen collection was 8 days, but the interquartile range shows a high variability, suggesting that the process was not always efficient.

Addressing this challenge requires a multi-faceted approach. First, additional resources, including staffing and equipment, are needed to expand the capacity for HCV screening and diagnosis within the prisons. Streamlining processes, such as implementing point-of-care RNA testing, could also improve efficiency and coverage [18, 19]. Second, establishing robust linkages with community-based healthcare providers is crucial to ensure continuity of care for participants released or transferred from prisons [19]. This would help to mitigate the loss to follow-up and ensure that these participants receive timely confirmatory testing and treatment. The implications of this bottleneck for resource allocation and sustainability are substantial. Without addressing this gap, the effectiveness of the prison-based HCV care model is significantly compromised. The high prevalence of HCV infection within the prison population necessitates a sustained effort to ensure that all HCV antibody-positive participants receive timely confirmatory testing and treatment.

On harm reduction services, it is important to reiterate that opioid agonist therapy (OAT) and needle and syringe programs (NSP) are not currently available within Kedah state prisons. However, HCV screening and treatment services are available outside the prisons at government primary health clinics and hospitals. This disparity in access to harm reduction services has implications for HCV transmission and care, both within and outside the prison setting. The high prevalence of people who inject drugs within the prison population, as shown in the risk factor analysis, further underscores the need for comprehensive harm reduction strategies.

Another advantage of the model is the same-day collection of blood samples for both HCV RNA testing and pretreatment assessment during the same prison clinic visit. This eliminates the need for repeated venipuncture, which is challenging for participants with difficult venous access. While this study indicates a longer time from RNA test results to treatment initiation, this still represents an improvement over the previous system prior to model implementation. This approach also reduces delays in obtaining results, facilitating a smoother transition into treatment. Additionally, this model employs a sequential combination of two noninvasive biomarkers to assess liver fibrosis severity [21], simplifying evaluation for nonspecialist medical staff in prisons. It eliminates the need for hospital-based ultrasound and associated logistical challenges, offering a practical solution in areas with limited equipment and expertise. However, it’s important to acknowledge that ultrasound may still be needed for screening for hepatocellular carcinoma, particularly in participants with advanced liver fibrosis.

In line with the government’s goal to expand treatment access beyond specialists, this model also empowered trained in-prison medical staff to initiate DAA-based therapy. These findings support national efforts to enhance treatment accessibility for participants at early stages of advanced liver diseases [30]. Apart from sofosbuvir-daclatasvir, the study enabled the use of sofosbuvir-ravidasvir, a combination newly added to the WHO Model Lists of Essential Medicines, in prisons [31, 32]. Sofosbuvir-ravidasvir is a pangenotypic DAA treatment that can be used for all eligible participants regardless of genotypes [32]. Participants receiving this regimen generally reported a favourable treatment response. An ongoing clinical trial is evaluating the efficiency and safety of shorter treatment durations with this combination (ClinicalTrials.gov ID: NCT04885855, registered: 13 May 2021), potentially expected to enhance treatment adherence and improve HCV care delivery in correctional settings.

Overall, participants receiving treatment in this study achieved a high SVR rate (96.3%), comparable to those reported in community settings [14, 22]. These findings strengthen confidence in the feasibility of this model and its effectiveness for broader implementation in correctional settings.

Despite its strengths, a significant challenge for the model lies in retention throughout the HCV care cascade. The study identified a substantial proportion of participants were released or transferred to other prisons at various stages, resulting in loss to follow-up and preventing the assessment of sustained virologic response (SVR12) in these participants. This loss to follow-up has adversely impacted treatment adherence, with dropouts observed during treatment initiation, throughout the treatment period, and before the SVR12 testing. Similar challenges with participant retention due to release or transfer have been documented in other prison settings [24]. In the current study, these dropouts may be partly due to the implementation of parole systems and earned remission policies aimed at alleviating prison overcrowding.

To address this challenge, a multipronged approach is necessary. Establishing stronger communication between prison health services and the court system would allow for a more informed decision-making process regarding parole or release, considering the participant’s treatment status and progress. Better communication could also enable more effective release planning to support treatment continuation after release. Additionally, developing protocols for transferring treatment information and medications to receiving facilities, whether within the prison system or in community settings, is crucial to ensure continuity of care and minimise the likelihood of dropouts. Strengthening connections with community health clinics upon release is also essential. Support groups, civil society organisation or patient navigators can further enhance treatment adherence and SVR achievement for these participants [33]. Future studies should explore strategies for maintaining contact with released participants to ensure treatment completion and assess SVR12.

This study found a higher treatment initiation rate among participants under detention than among those serving sentences. Although the exact reason for this difference is unclear, it is possible that the in-prison medical staff might be hesitant to initiate treatment for participants serving sentences, particularly those nearing release or actively seeking parole. Concerns about adherence and potential loss to follow-up after release could negatively impact the HCV care delivery. However, the dynamics of prison environments are complex and can influence treatment decisions in unforeseen ways. Factors such as the perceived stability of remand populations, the urgency of medical needs during detention, and the specific protocols within each prison could also play a role. Future research could explore these factors to develop targeted interventions and promote treatment initiation across different types of prisons.

It is also counterintuitive that those under sentences experienced a shorter wait time to start HCV treatment after a positive RNA test, even though these participants were less likely to be treated overall. A possible explanation is that when participants under sentences were deemed eligible and approved for treatment, the in-prison medical team prioritised their treatment initiation to ensure adherence and completion within their remaining sentence. This prioritisation could have resulted in a shorter wait time for those who were ultimately treated. Nevertheless, this is a post-hoc explanation, and further research is needed to explore the underlying factors contributing to this finding. Additionally, it is important to note that no significant differences were observed at other stages of the HCV care cascade between the two prisons. Furthermore, there were no significant differences in the total time from HCV antibody testing to treatment initiation between the two prisons. These findings suggest that the test-and-treat model is feasible regardless of prison type.

While possible reasons for the three participants who did not achieve SVR12 was unknown and could be due to either new infection or reinfection, this study did not collect behavioural data on transmission risk factors, including intravenous drug use (IDU), within the prisons, that could explain possible HCV reinfection. Global studies have indicated that drug use during incarceration is prevalent, particularly among individuals with a history of pre-incarceration drug use [34, 35]. Reinfection with HCV is also a potential concern, particularly in settings with ongoing risk behaviours such as within prison. Therefore, future investigations into the prevalence of IDU and other risk factors within Malaysian prisons are essential to better understand the risk of reinfection and inform targeted interventions. Given the potential for reinfection, extending prison entry test and treat models to include periodic screening post-treatment could be beneficial. Such screening could detect reinfection early, allowing for prompt retreatment and reducing ongoing transmission within the prison setting.

Some study limitations are worth highlighting. First, a significant number of missing information among the large proportion of participants who were anti-HCV positive but did not receive an HCV RNA test. As previously discussed, approximately 80% of HCV antibody-positive participants did not receive RNA testing, primarily due to limited capacity within the prison medical teams and participant unavailability due to release or transfer. Consequently, our analyses were primarily conducted among the subset of participants who received RNA testing. This missing information may limit the generalisability of our findings to the entire population of HCV antibody-positive participants. It is possible that those who did not receive RNA testing had different demographic and clinical characteristics, or outcomes compared to those who did. We have discussed the potential reasons for this missing information and its implications for the interpretation of our results in the previous section. Second, the study did not evaluate the implementation costs or compare costs across prison types. While funding is derived primarily from the annual allocation for the hospital [30], conducting a comprehensive cost analysis, including long-term financial implications, would enhance the model’s evaluation. Third, further exploration is needed to generalise the findings to other prison settings with diverse population densities and varying healthcare resource availability. Conducting studies that implement this model in geographically diverse prison settings would be beneficial.

In conclusion, this new prison-based test-and-treat model has demonstrated a promising approach for identifying and treating HCV infection within prisons. The collaborative effort involving multiple government agencies exemplifies a successful strategy for advancing HCV elimination efforts. The model integrates HCV testing with entry screening to maximise case detection and optimise existing resources, ensuring financial viability in resource-constrained settings. Despite major challenges in maintaining participant retention throughout treatment, access to HCV RNA testing and continuity of care for people released from prisons, the proposed solutions offer promising avenues for improvement. This study provides valuable evidence supporting the implementation of this new model in LMICs to address the burden of HCV infection among people who are in prisons and contribute to global elimination efforts.

## Data Availability

The datasets used and analysed during the current study are available from the corresponding author upon reasonable request.

## References

[1] World Health Organization. Global hepatitis report 2024: Action for access in low- and middle-income countries. 2024. https://www.who.int/publications/i/item/9789240091672. Accessed 8 Feb 2025.

[2] World Health Organization, Hepatitis C. Fact Sheet. 2023. https://www.who.int/news-room/fact-sheets/detail/hepatitis-c. Accessed 6 June 2024.

[3] Khatun M, Ray RB. Mechanisms underlying hepatitis C virus-associated hepatic fibrosis. Cells. 2019. 10.3390/cells8101249. 31615075 10.3390/cells8101249 PMC6829586

[4] Younossi Z, Park H, Henry L, Adeyemi A, Stepanova M. Extrahepatic manifestations of hepatitis C: a meta-analysis of prevalence, quality of life, and economic burden. Gastroenterology. 2016. 10.1053/j.gastro.2016.02.039. 26924097 10.1053/j.gastro.2016.02.039

[5] Stanaway JD, Flaxman AD, Naghavi M, Fitzmaurice C, Vos T, Abubakar I, et al. The global burden of viral hepatitis from 1990 to 2013: findings from the global burden of disease study 2013. Lancet. 2016. 10.1016/S0140-6736(16)30579-7. 27394647 10.1016/S0140-6736(16)30579-7 PMC5100695

[6] World Health Organization. Global health sector strategy on viral hepatitis 2017–2021. 2016. https://www.who.int/publications/i/item/WHO-HIV-2016.06. Accessed 13 July 2024.

[7] Martinello M, Naggie S, Rockstroh JK, Matthews GV. Direct-acting antiviral therapy for treatment of acute and recent hepatitis C virus infection: a narrative review. Clin Infect Dis. 2023. 10.1093/cid/ciad344. 37579203 10.1093/cid/ciad344

[8] Salama II, Raslan HM, Abdel-Latif GA, Salama SI, Sami SM, Shaaban FA, et al. Impact of direct-acting antiviral regimens on hepatic and extrahepatic manifestations of hepatitis C virus infection. World J Hepatol. 2022. 10.4254/wjh.v14.i6.1053. 35978668 10.4254/wjh.v14.i6.1053 PMC9258264

[9] Tang W, Tao Y, Fajardo E, Reipold EI, Chou R, Tucker JD, et al. Diagnostic accuracy of point-of-care HCV viral load assays for HCV diagnosis: a systematic review and meta-analysis. Diagnostics. 2022. 10.3390/diagnostics12051255. 35626411 10.3390/diagnostics12051255 PMC9141110

[10] Trickey A, Fajardo E, Alemu D, Artenie AA, Easterbrook P. Impact of hepatitis C virus point-of-care RNA viral load testing compared with laboratory-based testing on uptake of RNA testing and treatment, and turnaround times: a systematic review and meta-analysis. Lancet Gastroenterol Hepatol. 2023. 10.1016/S2468-1253(22)00346-6. 36706775 10.1016/S2468-1253(22)00346-6 PMC11810864

[11] World Health Organization. Guidelines for the care and treatment of persons diagnosed with chronic hepatitis C virus infection. Geneva: World Health Organization; 2018. 30307724

[12] Ministry of Health Malaysia. National strategic plan for hepatitis B and C 2019–2023. 2019. https://www.moh.gov.my/moh/resources/Penerbitan/Pelan%20Strategik%20/NSP_Hep_BC_2019_2023.pdf?gh_jid=4958715003. Accessed 13 July 2024.

[13] Kamarulzaman A, Reid SE, Schwitters A, Wiessing L, El-Bassel N, Dolan K, et al. Prevention of transmission of HIV, hepatitis B virus, hepatitis C virus, and tuberculosis in prisoners. Lancet. 2016. 10.1016/S0140-6736(16)30769-3. 27427456 10.1016/S0140-6736(16)30769-3

[14] Markby J, Shilton S, Sem X, Chan HK, Said RM, Siva S, et al. Assessing the impact of simplified HCV care on linkage to care amongst high-risk patients at primary healthcare clinics in Malaysia: a prospective observational study. BMJ Open. 2021. 10.1136/bmjopen-2021-055142. 34952885 10.1136/bmjopen-2021-055142 PMC8713014

[15] Castro R, Perazzo H, de Araujo LAMM, Gutierres IG, Grinsztejn B, Veloso VG. Effectiveness of implementing a decentralized delivery of hepatitis C virus treatment with direct-acting antivirals: a systematic review with meta-analysis. PLoS ONE. 2020. 10.1371/journal.pone.0229143. 32084187 10.1371/journal.pone.0229143 PMC7034833

[16] Calleja JL, Espin J, Kaushik A, Hernandez-Guerra M, Blissett R, Yehoshua A, et al. The efficiency of increased HCV testing and treatment strategies in Spain to achieve elimination goals. Pharmacoecon Open. 2024. 10.1007/s41669-023-00458-3. 38100074 10.1007/s41669-023-00458-3 PMC10884368

[17] Mera J, Williams MB, Essex W, McGrew KM, Boeckman L, Gahn D, et al. Evaluation of the Cherokee Nation hepatitis C virus elimination program in the first 22 months of implementation. JAMA Netw Open. 2020. 10.1001/jamanetworkopen.2020.30427. 33337496 10.1001/jamanetworkopen.2020.30427 PMC7749444

[18] Avramovic G, O’Doherty L, McHugh T, Remy AJ, Happiette A, Bouchkira H et al. Benchmarking of an intervention aiming at the micro-elimination of hepatitis c in vulnerable populations in Perpignan, France, to inform scale-up and elimination on the French Territory. Viruses. 2024; 10.3390/v16101645 10.3390/v16101645 PMC11512308 39459977

[19] Cabezas J, Llerena S, Mateo M, Álvarez R, Cobo C, González V, et al. Hepatitis C micro-elimination beyond prison walls: navigator-assisted test-and-treat strategy for subjects serving non-custodial sentences. Diagn. 2021. 10.3390/diagnostics11050877. 10.3390/diagnostics11050877 PMC8155928 34068955

[20] Ministry of Health Malaysia. Clinical practice guidelines: management of chronic hepatitis C in adults. 2019. https://www.moh.gov.my/moh/resources/Penerbitan/CPG/Gastroenterology/CPG_Management_of_Chronic_Hepatitis_ C_in_ Adults.pdf. Accessed 6 June 2024.

[21] Suan MAM, Chan HK, Sem X, Shilton S, Hassan MRA. Diagnostic performance of two non-invasive biomarkers used individually and in sequential combination for cirrhosis associated with hepatitis C virus infection. Sci Rep. 2022. 10.1038/s41598-022-24612-9. 36418369 10.1038/s41598-022-24612-9 PMC9684447

[22] Hassan MRA, Chan HK, Nordin M, Yahya R, Sulaiman WRW, Merican SAA, et al. Assessing feasibility of a modified same-day test-and-treat model for hepatitis C among rural people who inject drugs. Harm Reduct J. 2023. 10.1186/s12954-023-00800-2. 37046294 10.1186/s12954-023-00780-3 PMC10091347

[23] R Core Team. R: A Language and environment for statistical computing. Vienna, Austria: R Foundation for Statistical Computing; 2014.

[24] Papaluca T, McDonald L, Craigie A, Gibson A, Desmond P, Wong D, et al. Outcomes of treatment for hepatitis C in prisoners using a nurse-led, statewide model of care. J Hepatol. 2019. 10.1016/j.jhep.2019.01.012. 30654067 10.1016/j.jhep.2019.01.012

[25] Sheehan Y, Cunningham EB, Cochrane A, Byrne M, Brown T, McGrath C, et al. A ‘one-stop-shop’ point-of-care hepatitis C RNA testing intervention to enhance treatment uptake in a reception prison: the PIVOT study. J Hepatol. 2023. 10.1016/j.jhep.2023.04.019. 37116714 10.1016/j.jhep.2023.04.019

[26] Rumble C, Pevalin DJ, O’Moore É. Routine testing for blood-borne viruses in prisons: a systematic review. Eur J Public Health. 2015. 10.1093/eurpub/ckv133. 26219884 10.1093/eurpub/ckv133 PMC4668329

[27] He T, Li K, Roberts MS, Spaulding AC, Ayer T, Grefenstette JJ, et al. Prevention of hepatitis C by screening and treatment in U.S. Prisons. Ann Intern Med. 2016. 10.7326/M15-0617. 26595252 10.7326/M15-0617 PMC4854298

[28] Winter RJ, Sheehan Y, Papaluca T, Macdonald GA, Rowland J, Colman A, et al. Consensus recommendations on the management of hepatitis C in Australia’s prisons. Med J Aust. 2023. 10.5694/mja2.51854. 36871200 10.5694/mja2.51854

[29] Morris MD, Brown B, Allen SA. Universal opt-out screening for hepatitis C virus (HCV) within correctional facilities is an effective intervention to improve public health. Int J Prison Health. 2017. 10.1108/IJPH-07-2016-0028. 28914118 10.1108/IJPH-07-2016-0028 PMC5764160

[30] Chan HK, Hassali MA, Said RM, Hassan MRA. Treatment coverage and drug expenditure in hepatitis C patients from 2013 to 2019: a journey of improving treatment accessibility in Malaysia through government-led initiatives. Hepat Mon. 2020. 10.5812/hepatmon.107372.

[31] Cheong MWL, Piedagnel JM, Khor SK. Ravidasvir: equitable access through an alternative drug development pathway. Lancet Glob Health. 2021. 10.1016/S2214-109X(21)00357-0. 34678186 10.1016/S2214-109X(21)00357-0

[32] Andrieux-Meyer I, Tan SS, Thanprasertsuk S, Salvadori N, Menétrey C, Simon F, et al. Efficacy and safety of Ravidasvir plus Sofosbuvir in patients with chronic hepatitis C infection without cirrhosis or with compensated cirrhosis (STORM-C-1): interim analysis of a two-stage, open-label, multicentre, single arm, phase 2/3 trial. Lancet Gastroenterol Hepatol. 2021. 10.1016/S2468-1253(21)00031-5. 33865507 10.1016/S2468-1253(21)00031-5 PMC9767645

[33] Adda D, James C, Peck R, Ali M, Tiwana T, Kolawole T, et al. The role of nonprofit and nongovernmental organizations and people with viral hepatitis on the path toward hepatitis C virus elimination. J Infect Dis. 2023. 10.1093/infdis/jiad104. 37703339 10.1093/infdis/jiad104

[34] Norman C. A global review of prison drug smuggling routes and trends in the usage of drugs in prisons. WIREs Forensic Sci. 2023. 10.1002/wfs2.1473.

[35] Favril L. Drug use before and during imprisonment: drivers of continuation. Int J Drug Policy. 2023. 10.1016/j.drugpo.2023.104027. 37060886 10.1016/j.drugpo.2023.104027

